# The pulmonary effects of intravenous adenosine in asthmatic subjects

**DOI:** 10.1186/1465-9921-7-139

**Published:** 2006-11-30

**Authors:** Nausherwan K Burki, Mahmud Alam, Lu-Yuan Lee

**Affiliations:** 1 Division of Pulmonary & Critical Care Medicine, Department of Medicine, University of Connecticut Health Center, Farmington, CT, USA; 2 Department of Physiology University of Kentucky Medical Center, Lexington, KY, USA

## Abstract

**Background:**

We have shown that intravenous adenosine in normal subjects does not cause bronchospasm, but causes dyspnea, most likely by an effect on vagal C fibers in the lungs [Burki et al. J Appl Physiol 2005; 98:180-5]. Since airways inflammation and bronchial hyperreactivity are features of asthma, it is possible that intravenous adenosine may be associated with an increased intensity of dyspnea, and may cause bronchospasm, as noted anecdotally in previous reports.

**Methods:**

We compared the effects of placebo and 10 mg intravenous adenosine, in 6 normal and 6 asthmatic subjects.

**Results:**

Placebo injection had no significant (p > 0.05) effect on the forced expiratory spirogram, heart rate, minute ventilation (Ve), or respiratory sensation. Similarly, adenosine injection caused no significant changes (p > 0.05) in the forced expiratory spirogram; however, there was a rapid development of dyspnea as signified visually on a modified Borg scale, and a significant (p < 0.05) tachycardia in each subject (Asthmatics +18%, Normals + 34%), and a significant (p < 0.05) increase in Ve (Asthmatics +93%, Normals +130%). The intensity of dyspnea was significantly greater (p < 0.05) in the asthmatic subjects.

**Conclusion:**

These data indicate that intravenous adenosine does not cause bronchospasm in asthmatic subjects, and supports the concept that adenosine-induced dyspnea is most likely secondary to stimulation of vagal C fibers in the lungs. The increased intensity of adenosine-induced dyspnea in the asthmatic subjects suggests that airways inflammation may have sensitized the vagal C fibers.

## Background

The respiratory effects of adenosine, and endogenous nucleoside, have been studied in animals and man. Adenosine is also used therapeutically to treat supraventricular tachycardia [1]. Amongst the reported side effects [2] of intravenous adenosine in asthmatics are bronchospasm and dyspnea; however, in normal subjects we have shown [3] that while intravenous adenosine is dyspnogenic, and also stimulates ventilation and tachycardia, it does not cause bronchospasm. In rats we have shown that intravenous adenosine directly stimulates pulmonary vagal C fibers through activation of A_1 _receptors [4], and it is likely that the dyspnea in man is a direct consequence of pulmonary C fiber activation. Asthmatic subjects are known to have airways inflammation hyperreactivity and it is probable that airway vagal fibers are sensitized in these subjects.

We therefore studied normal subjects and asthmatics, to document whether the intensity of adenosine-induced dyspnea is altered in asthma and whether it is associated with bronchospasm.

## Methods

We studied six healthy normal subjects, and 6 subjects with mild to moderate persistent asthma [5]. They were all non-smokers and the asthmatic subjects were selected on the basis of a history of asthma [5], and airways obstruction (FEV_1_/FVC < 70%), with significant response (Δ FEV_I _> +15%) to inhaled bronchodilator. The asthmatic subjects (Mean age ± sd: 40.0 ± 11.0 years; three females) were all non-smokers. One subject had exercise-induced asthma and only used inhaled beta agonists prior to exercise, with irregular, occasional use of inhaled steroids; all the other subjects were on regular inhaled steroid treatment, with inhaled beta – agonists used either on a regular two to four times daily basis (2 subjects) or used as needed for symptomatic relief. None of the subjects had had any acute exacerbations of asthma within the previous 3 months. Baseline FVC was 97.0 ± 20.6% of the predicted value (range 69% – 119%), and FEV1 was 65.3 ± 12.2% of the predicted value (range 54% to 95%). All subjects were asked to refrain from using any beta-agonist drugs or caffeine containing beverages for 12 hours before the study day. Written informed consent as approved by the IRB was obtained from each subject.

Each subject was seated and a forearm vein was cannulated and connected to a normal saline drip. A curtain between the subject and the cannulated forearm prevented the subject from being able to see when an injection was given (see below). Ventilation, ventilatory pattern, and forced expiratory spirograms [6] were recorded with the subject breathing via a mouthpiece attached to a two-way valve (Hans-Rudolph, Kansas City, MO); expiratory flow was recorded on a multi-channel recorder (Grass Medical Instruments, Astro-Med Inc, West Warwick, RI) as the differential pressure signal from a heated pneumotachygraph on the expiratory side of the valve, connected to a differential pressure transducer (Hans-Rudolph, Kansas City, MO). The flow signal was electronically integrated to volume and recorded. The system was calibrated before each experiment, using a calibrated syringe (Spirometrix, Inc).

End-tidal CO2 was sampled at the mouthpiece through a needle attached to a CO2 meter (Ohmeda, Englewood, CO), the output from which was continuously recorded.

Arterial O_2 _saturation (SaO_2_) and heart rate were recorded continuously using a pulse oximeter (Criticare Systems, Inc. Waukesha, WI).

Measurements of dyspnea were made using a modified Borg scale [7] to which the subject pointed. The subjects were asked to focus on respiratory symptoms such as chest tightness, shortness of breath, increased urge to breathe, burning sensation in the chest and throat; preliminary studies had indicated that the commonest symptoms expressed were shortness of breath and chest tightness.

Each subject received either an injection of placebo (normal saline), followed by adenosine, 10 mg, or vice versa; the sequence was randomized, and subjects and observers were blinded to the sequence.

Baseline spirometry measurements were made and when the subject had a stable breathing pattern (as judged by <5% variation in the end-tidal CO_2_), minute ventilation (Ve), ventilatory pattern, end-tidal CO_2_, heart rate (H.R.), and SaO_2 _were recorded over three minutes, and a baseline Borg score was recorded.

The subject then received the injection of placebo or adenosine, care being taken to avoid awareness of the injection by the subject. Measurements of ventilation, end-tidal CO_2_, SaO_2 _and HR were made continuously over the next 15 minutes. Borg scores were recorded immediately after the injection and every 20 seconds thereafter for the next 5 minutes. Spirometry was measured at baseline, and at 3 minutes, 5 minutes, 10 minutes and 15 minutes post injection. Thirty minutes after the first injection, a second injection of either placebo or adenosine was given, and further measurements made for another 15 minutes.

Statistical analysis of the data within each group was performed by repeated measures one way ANOVA [8]. Comparison between groups was by two-way ANOVA [8]. Comparison of baseline values between the groups was performed by unpaired t test.

## Results

As expected, the FEV1, FVC, and FEV1/FVC values were significantly (p < 0.03) lower in the asthmatic versus the normal subjects (Table 1)

**Table 1:** Spirometry in Normal & Asthmatic Subjects (Mean ± SD)

	Normals						Asthmatics					
	
	Placebo			Adenosine			Placebo			Adenosine		
	
	Baseline	Post	p	Baseline	Post	p	Baseline	Post	p	Baseline	Post	p
FEV_1_, L	3.63 ± 0.49	3.61 ± 0.51	ns	3.63 ± 0.56	3.67 ± 0.59	ns	2.66 ± 0.75	2.61 ± 0.85	ns	2.68 ± 0.78	2.71 ± 0.84	ns
FVC, L	4.53 ± 0.65	4.48 ± 0.60	ns	4.47 ± 0.71	4.50 ± 0.72	ns	4.09 ± 0.88	4.02 ± 1.00	ns	4.09 ± 1.00	4.10 ± 1.00	ns
FEV_1_/FVC,%	80.0 ± 3.0	80.3 ± 3.4	ns	81.5 ± 3.6	83.5 ± 2.4	ns	65.3 ± 12.9	65.2 ± 14.5	ns	66.0 ± 12.7	55.0 ± 12.9	ns

Baseline and Post-bronchodilator values significantly different (p < 0.03) between normal and asthmatic subjects.

Neither saline injection nor intravenous adenosine resulted in any significant change (p > 0.5) in the spirogram (Table 1).

Placebo injection resulted in no significant (p > 0.5) change in Ve, ventilatory pattern, end-tidal CO_2_, SaO_2 _or HR, and no subject indicated any change in the Borg score.

**Figure 1:**
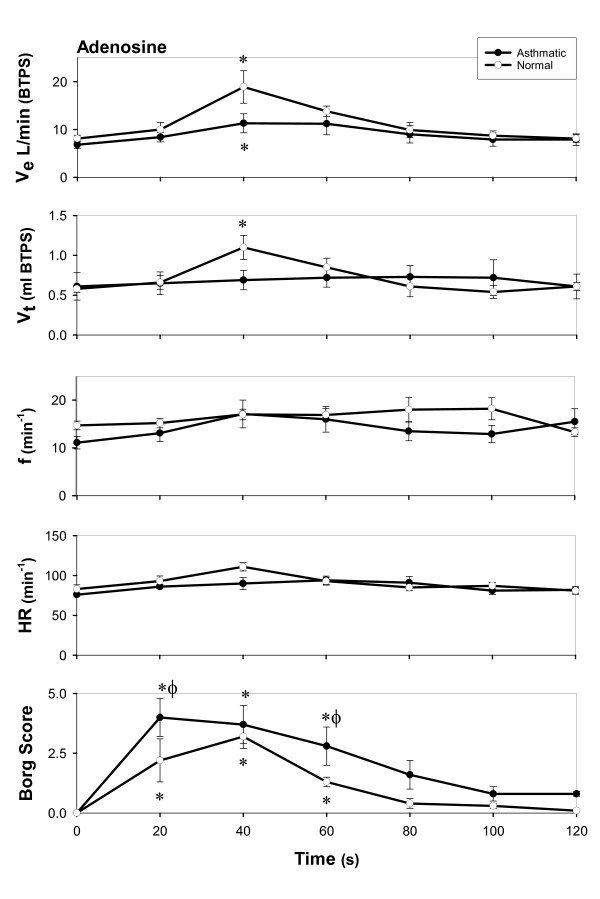
Effects of adenosine in normal and asthmatic subjects. Mean ± SEM. ○ = normal subjects, ● = asthmatic subjects. * = difference from baseline p < 0.05, repeated measures ANOVA φ = difference from Normals, p < 0.05, two way ANOVA

On the other hand, adenosine injection resulted in significant increases in Ve and heart rate in both groups (Figure 1).

All subjects experienced an increase in respiratory sensation after the adenosine injection, represented by an increase in the Borg dyspnea score (Fig 1). The changes in Borg dyspnea score were evident within 20–40 seconds, which was also the time of peak intensity of the dyspnea; in verbalizing the sensation after intravenous adenosine, 9 subjects described "chest tightness", 7 subjects described the sensation as "shortness of breath/difficulty in breathing" and 3 subjects described constriction in the throat. The increase in HR and Ve occurred slightly later, and peaked within the first 40–60 seconds after adenosine injection. The dyspnea, as well as the tachycardia and increased ventilation returned to baseline within 3 minutes of the injection. The initial intensity of the dyspnea was significantly greater (p < 0.05) and of longer duration in the asthmatic subjects (Figure 1).

## Discussion

The present study confirms our previous results in normal subjects [3] that intravenous adenosine is dyspnogenic and results in an increase in ventilation and tachycardia, and extends these observations to asthmatics.

Intravenous infusion of adenosine is known to increase heart rate by increasing cardiac sympathetic tone [1,9-11]; this effect overrides the bradycardia caused by its direct effects on the sinus node and atrio-ventricular conduction. In normal subjects we have shown [3] that adenosine causes tachycardia; the results of the present study are in conformity with these previous reports: intravenous adenosine was associated with a significant tachycardia.

The ventilatory effects of adenosine have previously been ascribed to activation of carotid chemoreceptors [9,11]; however, our previous data [3] indicate that the ventilatory effects of adenosine are probably not secondary to carotid chemoreceptor stimulation per se.

There are anecdotal reports [1,2] of bronchospasm in asthmatics receiving intravenous adenosine for the treatment of arrhythmia. However bronchoconstriction has never been documented by any measurements of airway function. In a study [12] of 122 consecutive patients, undergoing adenosine stress testing for myocardial perfusion imaging, of whom 36 had chronic bronchitis, dyspnea was noted in over 50% of subjects but no changes in spirometric indices were noted. Previous studies have found no change in airway function in normal subjects [1,3] after intravenous adenosine, and our results are in conformity with these findings, and further extend these observations to asthmatic subjects, since there was no significant change in the spirogram after adenosine. Thus it is probable that dyspnea in asthmatics receiving intravenous adenosine has been misinterpreted as bronchoconstriction in these instances.

Inhaled adenosine and adenosine 5-monophosphate and triphosphate are known to cause bronchospasm in asthmatics probably via mast cell mediator release [13-16] and, since this effect has not been demonstrated with intravenous adenosine [1,3,12], this suggests that the bronchospastic effect of adenosine is related to the route of administration. The bronchospasm occurring with inhaled adenosine monophosphate and triphosphate is also associated with the development of dyspnea; this has been interpreted as being secondary to the bronchospasm, although differences in the intensity of dyspnea for equivalent degrees of bronchospasm have been ascribed to an additional effect on airway sensory nerves [15,16].

Dyspnea, or shortness of breath [17-19], is a common accompaniment of most lung diseases, however, this sensation remains poorly understood and a number of theories have been put forth as to the genesis of this sensation [17,18]. The currently accepted view central, chemoreceptor, and peripheral (chest wall mechanoreceptor and lung receptor) mechanisms [18-20]. The present study examined the role of lung receptors in the genesis of dyspnea.

Sensory receptors in the lungs are innervated by the vagus nerves and consist of three types: stretch receptors and irritant receptors in the large airways, and unmyelinated vagal C fibers [21]. While the irritant receptors appear to modify the intensity of dyspnea associated with induced bronchoconstriction [22], and the airway stretch receptors appear to modify breathlessness by altering ventilatory pattern [23,24], neither of these receptors has been shown to be specifically dyspnogenic.

Vagal afferent C fibers have been implicated in the sensation of dyspnea [26]. These are small, unmyelinated nerve fibers that provide sensory input from airway and lung structures. These nerve endings are believed to lie in close proximity to the pulmonary capillaries and alveoli, and are also present in the bronchiolar epithelium of the conducting airways [21]. Pulmonary C fibers are now considered synonymous [25,26] with the "J" receptors described by Paintal [27] in the lung parenchyma. Some workers [21] have subdivided the C fibers into two groups, pulmonary and bronchial, whereas other investigators [28,29] have disputed this. It is also unclear whether there are differences in receptor properties between the two groups.

Stimulation of pulmonary vagal C fibers in animals results in apnea followed by tachypnea, airway smooth muscle contraction, mucus hypersecretion, and extravasation of macromolecules into the tracheobronchial tree [30]. In man, pulmonary C fibers have been implicated in the sensation of dyspnea [31], although direct evidence of this has been hard to obtain [32]. Human studies attempting to characterize C fibers have utilised intravenous lobeline or intravenous or aerosolized capsaicin; these produce coughing and burning or irritating sensations in the throat and midsternum [25,27,33-35]. These sensations are often so powerful that they limit the dose of the drug that can be administered. No attempt was made in these studies to differentiate the contributions from pulmonary and bronchial C fibers and the development of a cough raises the possibility that other receptors, such as the irritant receptors may also be stimulated by these drugs [25].

In normal human subjects, bilateral local anesthetic block of the vagus nerves at the base of the skull diminished breathholding sensation, and prolonged breathholding [36]; in dyspneic patients, bilateral vagal blockade reduced the intensity of dyspnea, but did not totally relieve the dyspnea [37,38]. In normal subjects, exercise-induced breathlessness is reduced, but not abolished, by local anesthetic blockade of alveolar receptors [32]. In contrast, in dyspneic patients with various lung diseases, local anesthesia of the airways did not modify the dyspnea, although the level and extent of airway anesthesia achieved is uncertain [39,40]. However, small particle aerosol-induced anesthesia of the smaller airways in rats abolished the tachypnea induced by microemboli, suggesting that vagal C fibers were involved in the tachypnea [32]. Evidence implicating a role for airway/alveolar C fibers in dyspnea comes from studies of nebulized morphine in dypneic patients [41] which reduced exercise induced dyspnea, and the finding of an increase in exercise induced dyspnea in normal subjects after inhalation of prostaglandin E2, which is known to increase vagal afferent C fiber sensitivity [42]. Thus, while previous evidence is suggestive, the results of studies of dyspnea and the role of pulmonary vagal C fibers have been inconclusive.

A study in rats in our laboratory [4] provided the first evidence that adenosine stimulates pulmonary C fibers through activation of A1 receptors. We extended these studies to normal human subjects [3,43] and showed that intravenous adenosine induces significant dyspnea in the absence of changes in airways resistance, and in association with an increase in ventilation. In these studies, we used hand grip dynamometry to assess respiratory sensation, and the interrupter technique for measuring airways resistance, which allowed us to closely examine the time latencies of these effects. We found that the dyspnogenic response was not related to the ventilatory or cardiac response and, as in the present study, preceded the ventilatory and cardiac responses. We concluded that the dyspnea is secondary to a direct effect of adenosine on vagal C fibers in the lung [3]. However, handgrip dynamometry does not allow comparison of the intensity of sensation between subjects, therefore in the present study we used the modified Borg scale and found that the intensity of dyspnea after intravenous adenosine was increased in asthmatics versus normal subjects.

The fact that the cardiac and ventilatory responses were significantly less in the asthmatic subjects in the present study, whereas the dyspnea rating had a greater intensity, lends further support to the concept that the dyspneic response is not related to the ventilatory or cardiac effects of adenosine and is probably not mediated by the carotid chemoreceptors, and supports the concept that it is most likely secondary to stimulation of vagal C fibers in the lungs. The increased intensity of dyspnea in the asthmatics may represent a lowered threshold of activation and/or a higher sensitivity of the C fibers, consequent to the airway inflammation in asthma.

Dyspnea is a cardinal symptom of asthma, but the precise mechanism of the dyspnea is unknown [44]. It has been shown [15] that dyspnea in asthmatics is directly related to the degree of airways obstruction in any given patient; however, between asthmatics, equivalent degrees of obstruction do not elicit equivalent degrees of dyspnea [15,16]. Furthermore, the intensity of dyspnea is directly related to the relationship between the minute ventilation and the central inspiratory neuromuscular drive: the lower this ratio, the greater the dyspnea [15].

It has been suggested [44] that dyspnea in asthma is due to a combination of the increased sense of effort secondary to the increased mechanical load, with an additional contribution from afferent receptors in the airways or lungs. Most workers have suggested that the latter are most likely the rapidly adapting irritant receptors in the airways [44-46]. However, these conclusions are based on the results of inhaled lidocaine blockade of the airways, although there is no way to assess from these studies whether there was also some partial blockade of the airway and pulmonary C receptors. Since airway inflammation is a cardinal feature of asthma [47] it is possible that inflammation stimulates the C fiber endings in the lungs and airways, resulting in dyspnea. The present study results would support such a conclusion.

In conclusion, this study has shown that intravenous adenosine results in dyspnea both in normal and asthmatic subjects, and that this effect is not related to bronchospasm. Adenosine also causes an increase in ventilation; however the dyspnogenic effect is clearly not a consequence of the increase in ventilation. Asthmatic subjects have a greater intensity of dyspnea in response to adenosine, perhaps secondary to sensitization of the airway vagal C fibers due to airways inflammation. The timing of the dyspnogenic response indicates that it is not related to carotid chemoreceptor stimulation and is most likely a consequence of direct stimulation of pulmonary vagal C fibers.

## Competing interests

The author(s) declare that they have no competing interests.

## Authors' contributions

N KB developed the hypothesis, and planned and performed the study. He is primarily responsible for drafting the final paper and the final results and conclusions of this study.

L-Y L assisted with developing the concepts and participating in the experiments and analysis of the results and conclusions. He was closely involved in developing and revising the draft of the manuscript.

MA was responsible with assisting in the experimental studies and the data analysis.

All authors read and approved the final manuscript.
